# Technical efficiency in milk production in underdeveloped production environment of India*

**DOI:** 10.1186/2193-1801-2-65

**Published:** 2013-02-23

**Authors:** Dwaipayan Bardhan, Murari Lal Sharma

**Affiliations:** 1 Department of Veterinary and Animal Husbandry Extension, College of Veterinary and Animal Sciences, G.B. Pant University of Agriculture and Technology, Pantnagar, Uttarakhand India; 2 Department of Agricultural Economics, College of Agricultural Sciences, G.B. Pant University of Agriculture and Technology, Pantnagar, Uttarakhand India

**Keywords:** Stochastic frontier analysis, Dairying, Uttarakhand

## Abstract

The study was undertaken in Kumaon division of Uttarakhand state of India with the objective of estimating technical efficiency in milk production across different herd-size category households and factors influencing it. Total of 60 farm households having representation from different herd-size categories drawn from six randomly selected villages of plain and hilly regions of the division constituted the ultimate sampling units of the study. Stochastic frontier production function analysis was used to estimate the technical efficiency in milk production. Multivariate regression equations were fitted taking technical efficiency index as the regressand to identify the factors significantly influencing technical efficiency in milk production. The study revealed that variation in output across farms in the study area was due to difference in their technical efficiency levels. However, it was interesting to note that smallholder producers were more technically efficient in milk production than their larger counterparts, especially in the plains. Apart from herd size, intensity of market participation had significant and positive impact on technical efficiency in the plains. This provides definite indication that increasing the level of commercialization of dairy farms would have beneficial impact on their production efficiency.

## Introduction

Despite of holding the number one position in global milk production, the milk productivity in India remains one of the lowest as compared to many leading countries of the world. At national level, milk yield of indigenous cow is about 3 to 3.5 litres, of buffalo 3.96 to 5.39 litres and of crossbred cow between 5.82 to 7.80 litres per day. As per FAO data, productivity of an average milch animal in India is even less than half of the world average. Productivity growth can be enhanced through two pathways – technological progress and technical efficiency improvement (Karanja et al. 2012). Technological progress requires substantial capital investment. In a developing country like India, it is important to know what policies and steps need to be taken for productivity enhancement before investing scarce capital to effect technological progress (Saha and Jain 2004). In this context, efficiency analysis assumes critical importance as technical efficiency improvement entails inefficient farmers adopting existing technologies and practices and thus saving scarce capital to get better results from them. Also, analysis of factors causing (in) efficiency offers crucial insights on key variables that might be worthy of consideration in policy making in order to ensure optimal capital and resource utilization.

Literature review has revealed that farmers in developing countries fail to exploit full potential of a technology and make allocative errors (Gelan et al. 2010; Otieno et al., 2012 and Rao and Rama 2012). Thus, increasing the efficiency in production assumes greater significance in attaining potential output at the farm level. Although several studies are available on analysis of technical efficiency in farm production in the Indian context (Narala and Zala 2010 and Mondal et al. 2012), studies on technical efficiency in milk production under mixed farming are rare (Saha and Jain 2004).

Efficiency analysis in milk production becomes all the more important in underdeveloped production environments of developing countries like India which are basically low-input and low-output environments characterized by subsistence holdings, resource poor locations with milch animals of low production potential and having poor infrastructural support system. In view of the above, the present study was carried out to examine the technical efficiency in milk production along with influence of various factors on this efficiency in Kumaon region of Uttarakhand state.

## Materials and methods

### Sample and data

The state of Uttarakhand has two divisions, viz. Kumaon and Garhwal. The study was confined in Kumaon division on account of greater livestock density in this division (114 per sq. km. geographic area and 840 per 1000 rural population as compared to 78 per sq. km. geographic area and 795 per 1000 rural population in Garhwal division) (Bardhan et al. 2010). Two districts rich in livestock resources, viz. U.S. Nagar (located in the plains) and Almora (located in hills) from Kumaon region were chosen so as to have a comparative picture of milk production scenario in the plains and hills. A total of three villages falling outside milk-routes of the dairy cooperative network were selected randomly from each district. Ten milk selling households were selected from each such village having representation from different herd-size categories, viz. small, medium and large (identified on the basis of Standard Animal Units using cumulative square root frequency technique) on proportionate basis. The final sample size comprised of 60 milk producing households. Data for the present study were collected through personal interview method with the help of a well-structured, comprehensive and pretested interview schedule.

### Estimation of technical efficiency in milk production

The objective of the present investigation was to estimate technical efficiency in milk production at household level. Therefore, it was very important to consider the effect of various species/breed of milch animals kept by farm households. For this purpose, Standard Animal Units (SAU) as per specifications given by Kumbhare et al. (1983) was derived to standardize output of different farms with different species of dairy animals^a^. The output variable was taken as fat corrected milk (FCM) production per SAU per day in litres based on the following formula as prescribed by Hemme (2000):

The two most popular approaches to estimation of technical efficiency are the parametric stochastic frontier analysis (SFA) and non-parametric Data Envelopment Analysis (DEA). Each of the two approaches has its own strengths and weaknesses. While the advantage with DEA lies in its general non-parametric frontier, its limitations are related to the fact that it attributes all deviations from the frontier to inefficiency ignoring stochastic noises in the data. On the other hand the strength of SFA lies in its ability to segregate the error term into two components, viz. inefficiency and statistical noise, but it can be implemented only by imposing a specific functional form and hence the efficiency indicators obtained can be sensitive to chosen function form (Gelan et al. 2010). It has been suggested in earlier works that SFA with a composed error term is more appropriate to estimate technical efficiency in agriculture, particularly in the developing countries, where the probability of the data being influenced by measurement errors and the effects of weather conditions, disease, etc. are high (Kumar et al. 2004). The present study used the stochastic frontier production function to estimate the technical efficiency in milk production in the study area. The stochastic frontier production function can be written in the general form as:

Where, Y_k_ is the output of the k^th^ farm, X_i_ ‘s are the inputs to the production process, v_k_ is a random variable representing statistical noise and other stochastic shocks entering into the definition of the frontier. It is almost universal to specify this random term as independent normally distributed with zero mean and constant unknown variance σ_v_^2^, and independent of X_i_, i.e. v_k_ ~ N (0, σ_v_^2^). u_k_ is a non-negative random variable representing technical inefficiency and is assumed to be distributed independently of v_k_ and X_i ._ It can be measured by the difference between maximum output Y* (estimated through the stochastic frontier production function) and observed output, Y_i_. Thus, farm-specific inefficiency is the distance below the frontier (Y_i_ – Y*). The above stochastic frontier production function can be estimated by maximum likelihood once a density function for u_k_ is specified.

Since, the input-output relationship in this study has been explored at household level and not for individual species of animals, some of the important variables such as order of lactation of milch animals and stage of lactation of milch animals have been purposively eliminated due to difficulty incorporating these information at an aggregated milk production function. Hence, it was assumed that the eliminated variables were not significantly varying between farm households in the study area (Saha and Jain 2004).

To take care of variations in the type of fodder fed at different times and the mixture of fodder fed; the feed inputs were standardized to nutrition units in terms of a feed index developed from Digestible Crude Protein (DCP) and Total Digestible Nutrients (TDN) content in the feeds and fodder. The estimates of feed index were worked out for the feeds and fodder for individual farms using the formulae DCP + (TDN/7.5). The value of family labour was imputed upon the prevailing wage rate in the study area. This was measured by collecting information regarding the amount of time spent by different household members in various activities related to dairying, and converting them into equivalent mandays.

### Factors influencing technical efficiency

Multivariate regression equations were fitted pooled over different categories of households, taking technical efficiency index as regressand and a set of variables representing farm and farmer characteristics and transaction cost variables as regressors. To control for the effect of herd size in the pooled equations, herd size was included as an additional independent variable in the final model. Multicollinearity among independent variables was tested by preparing a zero-order correlation matrix, for both the data sets, i.e. for plains and hills, separately. The specific variables used in this study as regressors in the regression equations are described in the Table 1.

**Table 1:** **Variables considered in the study**

Variables	Description	Measurement
Education-HH head	Education level of household age	0-Illiterate, 1-Read & write, 2-Primary, 3-Middle, 4-High school, 5-Intermediate, 6-Graduation & above
Age-HH head	Age of household head	Number of years
Landholding	Size of landholding of household	Hectares (ha)
Herd size	Number of milch animals owned by household	Measured as standard milch animal units
Non-farm income	Whether a household member has non-farm income source	1 = Yes
		0 = No
Prop. of output sold	Percentage of milk produced sold	%
Price received	Weighted average price received for each lire of milk normally sold	Rupees
		(In several instances, milk of different species and breeds like crossbred cow, indigenous cow and buffalo was sold by individual producer. Thus, the weighted average price of milk, taking quantity of each type of milk as weights, was considered in the study.)
Access to Information	Whether has easy access to information	1 = Yes
		0 = No

## Results and discussion

### Socio-economic profile of respondent households

Table 2 presents the socio-economic characteristics of respondent households. No significant difference was observed in age and farm experience of household heads in the plains and the hills. However, education level of household heads was significantly higher in the hills than in the plains (P < 0.10). Household heads on an average in the plains had studied up to primary school level, while their counterparts in the hills had studied on an average up to middle school level. Significantly greater proportion of farmers (70%) in the hills relied solely on agriculture for their livelihood as compared to the plains (33%). Animal husbandry was pursued as a subsidiary source of income by vast majority of farmers in both the plains and hills (83% and 63%, respectively). Significantly greater proportion (P < 0.05) of households (70%) in the hills had at least one member with non-farm income than that in the plains (40%). Incidence of migration was significantly higher (P < 0.01) in the hills (37%) as compared to the plains (7%). Average size of landholding was significantly higher (P < 0.05) in the plains (2 acres) than in the hills (0.82 acre). Average herd size was also significantly higher (P < 0.10) in the plains (3 SAU) than in the hills (2 SAU). There were no significant differences between the hills and the plains in regard to average distance from farm to nearest animal health centre and access to credit. However, average distance to market was significantly higher (P < 0.05) in the hills (8 km) than in the plains (5 km). Access to information was also significantly greater (P < 0.10) for households in plains than in the hills.

**Table 2:** **Socio-economic profile of respondent households**

Particulars	Plains	Hills
**I. Farmer Characteristics**		
*Age-Household Head (No. of yrs.)*	48.56 ± 10.44	50.82 ± 14.51
*Education-Household Head*^#^*	1.78 ± 1.77	2.79 ± 1.97
*Farm Experience-Household Head (No. of yrs.)*	39.72 ± 10.36	41.96 ± 13.70
Main occupation of HH Head (% of respondents surveyed in each category)***		
*Agriculture*	33.33	70.00
*Agricultural Labour*	23.33	3.33
*Private Job*	10.00	3.33
*Government Service*	-	13.33
*Business*	10.00	6.67
*Animal Husbandry*	16.67	-
*Pensioner*	6.67	3.33
Subsidiary Occupation of HH Head*		
*Agriculture + Animal Husbandry*	-	33.33
*Business*	10.00	-
*Animal Husbandry*	83.33	63.33
*Animal Husbandry + Others*	-	3.33
**II. Household Characteristics**		
*Family Size (Adult Equivalents)*^*##*^	4.34 ± 1.40	3.80 ± 1.08
Household with at least one member having non-farm income (% of respondent Households)**	40.00	70.00
Households with at least one member who has migrated elsewhere for earning income (% of respondent HH’s)***	6.67	36.67
**III. Farm Characteristics**		
Operational Land-holding (acres)**	2.01 ± 3.48	0.82 ± 0.77
*Land used for Dairying (acres)*	0.16 ± 0.26	0.00 ± 0.00
Dairy Animal Holding (Standard Animal Units)*	2.86 ± 2.45	1.79 ± 0.59
**IV. Institutional Support Structure**		
Distance to nearest AHS centre (km)	5.47 ± 2.33	5.29 ± 0.66
Access to Credit (1 = Yes, 0 = No)	0.06 ± 0.24	0.01 ± 0.22
Has insured milch animals (1 = Yes, 0 = No)	0.00 ± 0.00	0.00 ± 0.00
Distance to market (km)**	5.39 ± 1.72	7.86 ± 7.82
Is arranging transportation a problem (1 = Yes, 0 = No)	0.06 ± 0.02	0.29 ± 0.46
Whether has easy access to information (1 = Yes, 0 = No)*	0.95 ± 0.17	0.50 ± 0.51

^#^ 0-Illiterate, 1-Read & write, 2-Primary, 3-Middle, 4-High school, 5-Intermediate, 6-Graduation & above.

^##^ 4 children = 3 adult women = 2 adult men.

Significantly different at ***1%, **5% and *10% level of significance.

### Milk production patterns

Table 3 presents the milk production per household and milk yield per SAU for each her size category in both the regions. Milk production and milk yield were both significantly higher in the plains than in the hills. Average milk production and milk yield across all households in the plains were 25 litres/household/day and 8 litres/SAU/day, respectively, while the same figures for hills were 8.5 litres/household/day and 6 litres/SAU/day, respectively.

**Table 3:** **Milk production patterns (Per day)**

Herd size categories	Milk production	Milk yield
	plains	
Small	12.84^a^	9.45^a^
Medium	17.91^b^	5.68
Large	74.75	11.36
Overall	24.85^c^	8.51^b^
	**Hills**	
Small	7.69^a^	6.04^a^
Medium	10.91^b^	4.74
Large	-	-
Overall	8.38^c^	5.76^b^

Differences between figures - pertaining to a particular parameter - having same superscripts across same herd-size categories over plains and hills are significant up to 10% level of significance.

Table 4 elicits the herd-size category wise contribution to total milk produced and marketed in the study area. In the plains, large category households contributed the highest share to total milk production (48%) followed by small (29%) and medium (23%) categories. Share of large category households (56%) was also significantly larger than small (22%) and medium (21%) categories to total volume of milk marketed. In the hills, the contribution of small category households (72%) to total milk produced was significantly higher than that of medium category households (28%). Share of small category households (62%) to total milk marketed was significantly higher than that of medium category (38%) households. Thus, comparison of herd-size wise milk production and marketing in the hills and plains revealed significant differences. In the plains, large category households were the dominant category in terms of share in both milk production and marketing, while in case of the hills, the small category was the most dominant.

**Table 4:** **Herd-size wise contribution to total milk production and marketing**

Contribution to production	Plains	Hills
Small	28.88	72.10
Medium	23.33	27.90
Large	47.78	-
**Contribution to marketing**		
Small	22.26	62.35
Medium	21.34	37.65
Large	56.41	-

### Frontier functional analysis for milk production

The level of technical efficiency of a particular farm is characterized by the relationship between observed production and some ideal or potential production. The measurement of farm-specific technical efficiency is based upon deviation of observed output from the best production or efficient production frontier. If a farm’s actual production point lies on the frontier, it is perfectly efficient. If it lies below the frontier, then it is technically inefficient, with the ratio of the actual to potential production defining the level of efficiency of the individual farm. Stochastic estimations incorporate a measure of random error. This involves the estimation of a stochastic production frontier, when the output of a farm is a function of a set of inputs, inefficiency and random error. Maximum likelihood estimation (MLE) technique was employed to estimate the parameters of the Cobb-Douglas production function using Frontier 4.1 version software package.

The results for the same are presented in Table 5. The generalized likelihood ratio (LR) statistic for testing the null hypothesis for the absence of inefficiency effects in the Cobb-Douglas stochastic frontier production were 9.788 and 10.86 in case of plains and hills, respectively. The calculated LR statistics were statistically significant in both the cases, implying that the null hypothesis that there were no technical inefficiency effects in the Cobb-Douglas stochastic production function was rejected. The estimates of the gamma values of 0.979 and 0.949, respectively for plains and hills, were all statistically significant. Saha and Jain (2004) had reported a relatively lower gamma value (0.723) from their study on milk production efficiency in Haryana. The high levels of gamma values in this study indicate that inefficiencies in individual farms explained very high proportion of variations in milk yield. The statistical significance of the gamma values also indicates that the frontier models were significantly different from the OLS models or the deterministic frontier, in which there were no random errors in the production function.

**Table 5:** **Maximum likelihood estimates of stochastic cobb-douglas frontier milk production functions of standard animal units of households**

Variables	Plains	Hills
	MLE estimates	MLE estimates
Constant	0.641	0.194
	(0.162)	(0.668)
Depreciation	0.183	0.219
	(0.149)	(0.361)
Veterinary expenditures	0.137	0.009
	(0.147)	(0.022)
Miscellaneous expenditures	1.628*	0.287
	(0.915)	(0.406)
Green fodder index	0.055	−0.020
	(0.300)	(0.042)
Dry fodder index	0.065*	0.742
	(0.031)	(0.911)
Concentrate index	−0.062*	−0.248
	(-0.032)	(0.277)
Family labour	−0.125**	0.229
	(0.050)	(0.744)
γ = s^2^u / s^2^s	0.979***	0.949***
	(0.007)***	(0.554E-05)
LR test of the one-sided error	9.788***	10.86***
Log-likelihood function	21.13	31.82

Figures in parentheses indicate standard errors.

Significant at ***1%, **5% and *10% levels of significance.

In the plains, concentrate was found to be a significant factor negatively influencing milk production in MLE model, implying excessive feeding of concentrates to the dairy animals. The effect of dry fodder was on the other hand significant and positive implying that there is scope for profitably increasing the level of dry fodder fed to the animals. Miscellaneous expenditures also exerted significant and positive influence on milk production, implying suboptimal expenditures on miscellaneous items. Family labour was a significant variable and the effect was negative, implying that there is scope for curtailment in labour hours devoted to taking care of animals. Depreciation, veterinary expenditures and green fodder did not have significant influence on milk production. In case of the hills, no variable was observed to exert significant influence on milk production.

### Estimation of technical efficiency

To determine the technical efficiency of the different herd-size categories of households across groups, the mean technical efficiency indices of milk production for different sample farms were obtained. The indices of mean technical efficiency of farm households are presented in Table 6. The mean efficiency of households in plains and hills were almost same (90.73 and 89.27, respectively). Small farmers were the most efficient in the plains (mean efficiency of 94.57), followed by large (mean efficiency of 92.62) and medium famers (mean efficiency of 84.40). In the hills also, small farmers were more efficient (mean efficiency of 90.31) than their medium counterparts (mean efficiency of 85.49).

**Table 6:** **Mean technical efficiency estimates and increasing efficiency potential of different herd-size category households**

Herd-size categories	Plains		Hills	
	Mean technical efficiency	Mean potential to increase efficiency	Mean technical efficiency	Mean potential to increase efficiency
Small	94.57	5.34	90.31	8.62
Medium	84.40	14.62	85.49	14.01
Large	92.62	6.81	-	-
Overall	90.73	9.18	89.27	10.01

Based on the technical efficiency of the most efficient farm in each herd-size category, the average potential to increase milk production was determined. The potential for technical efficiency improvement of milk production in terms of reducing milk production costs was higher for medium and large farms (14.62% and 6.51%, respectively) than that for small farms (5.34%) in the plains. Overall – for all categories of households - if the average farmer was to achieve the efficiency level of its most efficient counterpart, then he would realize a 9.18 per cent cost saving.

Mean potential to increase efficiency for small and medium category farmers in the hills were 8.62 per cent and 14.01 per cent. Mean potential to increase efficiency for overall category was 10.01 per cent. This implies, that if the average farm in the hills was to achieve the technical efficiency level of the most efficient farm, then the average farm would realize an 10.01 per cent cost saving.

### Factors influencing farmers’ technical efficiency in milk production

Table 7 elicits the results of the multivariate linear regression analysis carried out to identify the significant factors influencing technical efficiency of dairy farms, in the plains and hills. Pooled regression equations across all herd-size categories over groups were fitted in case of both the plains and the hills. A variable herd size was included in the set of explanatory variables to control for different her size categories of households.

**Table 7:** **Factors influencing technical efficiency of farms**

Variables	Plains		Hills	
	**β**	**SE**	**β**	**SE**
Constant	0.792**	0.317	0.925***	0.168
Education-HH head	−1.82E-02	0.015	5.423E-03	0.010
Age-HH head	−3.06E-03	0.003	−2.54E-03*	0.002
Land holding size	3.574E-03	0.009	8.334E-03	0.023
Herd size	−3.10E-02**	0.013	1.174E-02	0.043
Non-farm income (Y = 1; N = 0)	0.117	0.064	−1.35E-02	0.050
Prop. of output sold	0.291**	0.129	−7.50E-02	0.108
Price received	−1.33E-03	0.015	4.68E-03	0.005
Access to Info. (Y = 1; N = 0)	0.149	0.106	1.47E-02	0.037
R^2^	0.597		0.398	
F-value	1.616		1.572	

Significant at ***1%, **5% and *10% levels of significance.

In the plains, the variable herd size significantly and negatively influenced technical efficiency indicating that farms with smaller herd size are more efficient in milk production. This finding is consistent with that obtained from mean technical efficiency analysis in the earlier section. Proportion of output sold had significant but positive influence on milk production efficiency implying that farmers with higher degree of intensity of market participation were more efficient in milk production. In the hills, only the variable age had significant influence on technical efficiency in milk production. The effect of age was negative implying that households with younger heads were more efficient in milk production. No other variables were found to impact level of technical efficiency, significantly.

## Conclusion

The study was set out to measure and explain technical efficiency in milk production under mixed farming system in Kumaon division of Uttarakhand, which represents an underdeveloped production environment of India. Stochastic Frontier Analysis was used to estimate the technical efficiency scores. The study revealed that variation in output across farms in the study area was due to difference in their technical efficiency levels. Majority of farmers in the study area use available technology sub-optimally and produce less than potential output. The mean technical efficiency was found 91 per cent among the sample households in the plains and 89 per cent among the sample households in the hills. Further it was found that smaller herd size and higher level of commercialization contribute positively to efficiency in the plains and lower age of household heads contribute positively to efficiency in the hills. Thus, for technical efficiency improvement the policy makers should focus on households with larger herd size in the plains and households headed by older family members in the hills to enable them to utilize the potential of existing technologies more efficiently. Further, strong and effective linkage of farms to market would provide incentives towards increasing their efficiency in production and thus realize substantial cost savings, especially in the plains.

## Endnotes

^a^The following standards were used to standardize herd size of the farm households:

Milch buffalo 1.30

Milch Crossbred cow 1.40

Milch Indigenous cow 1.00

## Authors’ information

Dwaipayan Bardhan: Part of Ph.D. Thesis of first author.
